# 3+ parity in Egypt: a multilevel decomposition of wealth-based inequality

**DOI:** 10.1186/s12889-020-08793-4

**Published:** 2020-05-12

**Authors:** Zeinab Khadr

**Affiliations:** 1 Department of Statistics, Faculty of Economics and Political Science, Cairo University, Giza, Egypt; 2 The Social Research Center, The American University in Cairo, New Cairo, Egypt

**Keywords:** Multilevel analysis, Decomposition, Egypt’s fertility, concentration index, Individual and area level attributes

## Abstract

**Background:**

Wealth disparities in levels of fertility are well documented in Egypt. Data from the Egypt Demographic and Health Survey (2014) showed that 61% of births to mothers from the poorest wealth quintile were of the third order or higher compared to 33% among mothers from the richest wealth quintile. The current paper investigates the main individual and socio-contextual level determinants of having more than two living children among women aged 30 and older, and decomposes its wealth-based inequality.

**Methods:**

The secondary analysis was based on the 2014 Egypt Demographic and Health Survey. Multilevel linear regression was used to model the number of additional living children a woman has after her first two living children. A decomposition analysis of the wealth-based concentration index was applied using a multilevel model.

**Results:**

Individual level and area level attributes explained 83 and 17% of the variation in 3+ parity, respectively. Only areas not well served by the health system were significantly related to 3+ parity.

Decomposition of the wealth-based concentration index revealed that 55.7 and 44.3% of the 3+ parity inequality were attributed to individual level attributes and area level attributes, respectively. At the individual level, early marriage accounted for 26% of the inequality in 3+ parity inequality. At the area level, living in areas not well served by the health system accounted for 22.3% of the 3+ parity wealth- based inequality, while highly gendered areas contributed 5.8% to this inequality. Areas’ random effects contributed 7.1% to the 3+ parity inequality, assist in identifying specific areas that require targeted policies.

**Conclusion:**

Multilevel decomposition allowed the contributions of both the individual and area level attributes to be quantified. The decomposition highlighted the need for more tailored policies that target different social groups and different areas. Decomposition analysis also pinpointed specific areas that require additional targeted policies. This targeted approach can be used to support the efficient use of limited resources within any society.

## Background

Extensive research efforts have attempted to trace health inequalities to people’s unfair access to social, economic and cultural resources and opportunities, which have been shaped by structural social factors that lie beyond the health system [1]. These research efforts expanded from modeling the relationship between health and its social determinants at the individual level to adding socio-contextual level determinants. While many of these research efforts in health modeling retained their simple single-level modeling, others recognized the effect of socio-contextual factors clustering on individuals and implemented the appropriate multilevel analysis [2–4]. Other research efforts focused on decomposition of health inequality into inequalities in its social determinants. This type of research focused mainly on decomposing health inequality into the inequalities in individual level attributes. Only a few studies incorporated contextual effects in the decomposition analysis [5]. However, these studies used dummy variables to represent the communities in which individuals live and implemented single-level health modeling. This simple approach to dealing with the socio-contextual factors misses the mark in identifying the main community level attributes that contribute to health inequality, as well as failing to take into consideration the clustering effect of the communities’ attributes on their members. The current study proposes the use of a combination of multilevel analysis with decomposition analysis in the study of health inequality. This type of analysis can provide more insights about the accurate contribution of the social and structural factors inequalities and their translation into inequalities in the area level attributes that effect this health issue. The proper identification of these contributions can lead to efficient use of limited resources in tackling health inequalities.

The current study attempts to fill this void in the research. The study implements a multilevel model and a decomposition analysis to investigate the additional number of children born to women with two living children in Egypt. Three area level attributes are developed to capture socio-contextual attributes and health system performance in the different areas of Egypt surveyed. To take into consideration the clustering of the individuals within the different areas, the analysis uses the multilevel regression technique.

## Fertility levels and 3+ parity in Egypt

Egypt is one of the most populous countries in the Middle East and Africa. According to the latest census (2017), the total population is 94.8 million persons, with an annual growth rate of 2.54% during the inter-censual period (2006–2017) [6]. By 2030, UN projections estimate the total population of Egypt will reach 120.8 million [7]. This large population can no doubt impede economic development and exert significant pressure on already strained resources needed for the health, education and wellbeing of the Egyptian population [8]. The rapid population growth currently observed in Egypt is a product of the fertility trajectory during the period 1995 to 2014. After a significant decline in the total fertility rate (TFR) between 1995 and 2005, from 3.6 to 3.1 children per woman, the TFR stagnated at 3 children per woman for almost 3 years. Between 2008 and 2014, the TFR reversed direction and started increasing, reaching 3.5 children per woman by 2014 [9]. However, in the absence of recent comprehensive fertility data, there is some evidence of a fertility decline between 2014 and 2018. The Central Agency of Public Mobilization and Statistics (CAPMAS) recorded a 12.5% decline in the number of births, from 2.72 million births in 2014 to 2.38 million births in 2018 [10].

Investigations of the factors underlying this fertility trajectory in Egypt reveal that an interplay of education, wealth and age at first marriage have shaped it [11–13]. Under-privileged segments of the population, defined either in terms of low standards of living, rural residence or low education, experienced a decline in fertility between 1995 and 2006. This decline was brought about through rising age at first marriage and declines in their fertility preference [11–13]. For the privileged segments of the population, El Tigani (2003), in a study of stalled fertility in Egypt, revealed that the rise in age at marriage for these segments had “played out” between 1995 and 2000, and the only avenue for fertility declines is through declines in marital fertility [11]. However, by 2000, these segments of the population were seeing an increase in fertility levels, which continued until 2014. This rise was the product of a decline in age at marriage [11, 12] due to wide spread education among young women [14]. By 2014, the fertility levels of the privileged and unprivileged segments of population were similar [13]. By 2014, education lost its impact on fertility and age at marriage and fertility preference became the main factors underlying fertility levels in Egypt [13]. The wide spread of education among young women combined with the limited work opportunities for women led educated women to early marriage [8].

In addition to explaining the fertility trajectory in Egypt, researchers also investigated fertility preference, examining both the main attributes of the couples who embraced the two-child family ideal [15–17] and the attributes of those to progress from two to three children [12]. Literature on this issue has identified privileged women (urban residents, highly educated women and those with work experience before marriage) as the most likely to profess a preference for the two-child family ideal. For a more elaborate investigation of the two-child family idea, El Zeini (2008) proposed a sequential three-stage framework of acceptance, preference and achievement for the replacement fertility [17]. In her work, El Zeini acknowledged the effect of both the individual attributes and the social-contextual environment in which women live on embracing and achieving the two-child family idea. She showed that acceptance of the replacement level fertility/two-child family ideal was strongly related to the perception of children’s cost relative to their benefits. By contrast, the perception of women that they have less control over their childbearing and/or fear side effects of contraceptives can diminish their acceptance of the two-child family ideal. For moving from acceptance to preference, egalitarian gender values were found to enhance preference for a two-child family ideal. Again, costs associated with fertility control and women’s perception of lack of control over childbearing had a dampening effect on their preference for a small family ideal. For the previous two stages, the two contextual factors of living in Upper Egypt (the most conservative region in Egypt), and/or in communities less supportive of family planning, decreased women’s likelihood to accept or prefer the two-child family ideal. The third stage of achievement showed that among those who indicated a preference for the two-child family ideal, low social status and cost of fertility regulation decreased women’s likelihood to achieve their preference of a two-child family ideal.

While the previous literature on fertility and its trajectory in Egypt was able to identify the major role played by education, age at marriage and contextual factors assessed in terms of place of residence (urban versus rural, or by region), differences in the mean number of living children by wealth continue to persist in Egypt. Data from the Egypt Demographic and Health Survey (2014) showed that more than 44% of the new births over the 5 years preceding the survey were of the third order, or more with significant wealth disparities [7]. Among mothers from the poorest quintile, about 61% of births were of the third order or more, compared to 33% among women from the richest quintile. Similar wealth disparities were also observed in the total number of children ever born. Women in the poorest quintile had 3.5 children on average compared to 2.3 for women in the richest wealth quintile. A recent report examining the priorities for reproductive health issues and their inequities in Egypt confirmed the significance of these disparities. The report showed that grand multiparity (having five or more children) was one of the major reproductive health and reproductive health inequity priorities in Egypt [18].

Previous research efforts focused their investigations on changes in overall fertility levels, the achievement of the replacement level or the transition to the third child. In their investigations, researchers focused on the individual level factors and implemented limited operationalization of the socio-contextual factors. The current research moves the discussion of high parity in Egypt to understanding the main factors underlying each additional child beyond the replacement level, with more succinct operationalization of the socio-contextual factors. It also assesses the effect of the main factor currently affecting Egypt’s fertility rate, namely age at marriage, within different social contexts. It further expands the discussion to assess wealth-based inequality in high parity in Egypt and the contribution of the inequality in the factors underlying it To the researcher’s knowledge, the effect of both the individual and the socio-contextual factors underlying high parity, their wealth-based disparities and their contribution to the overall wealth-based inequality of high parity has not yet been investigated within the Egyptian context.

The current paper investigates the main individual and socio-contextual level determinants of the number of additional children born beyond the replacement fertility level (two living children) and decomposes its wealth-based inequality. The results of the current analysis can assist in the identification of the points of intervention, both at the individual level and at the socio-contextual level, to reduce high levels of fertility, particularly among poor women.

## Methods

### Data source and study variables

The sample for the current study is based on data from the last round of the Egypt Demographic and Health Survey (EDHS) in 2014. The EDHS is a multi-round survey carried out periodically to investigate different population and reproductive health issues in Egypt including fertility, contraceptive use, infant and child mortality, maternal and child health immunization, nutrition, as well as health-related knowledge and attitudes. The dataset for 2014 had a total sample of 20,460 ever-married women between the ages of 15 and 49 with a response rate of 98.4% (97.6% in urban areas and 99.2% in rural areas) [9]. The survey implemented a three-stage sampling design (for more detailed information on the sampling techniques and data collection see [9]).

The current study uses a subsample of all women aged 30 years and older who have two living children. With a median age at marriage of 20.8 years and an average of 2.05 children for ever-married women aged 25–29 and 2.8 children for women aged 30–34 [9], limiting the analytical sample to women in the age range of 30 years and older provides women with the time to have their first two living children and to consider and/or implement their plans to have more children. These parameters of age and number of living children yielded an analytical sample of 11,601 ever-married women.

To assess the effect of the socio-contextual factors, the study subsample used the 25 Egyptian governorates covered by the 2014 EDHS with their sub-classification into urban and rural areas as its area level classification. With three governorates (Cairo, Port Said and Suez) classified as urban governorates, the final number of areas used in the analysis was 47.

Weights provided by the EDHS datasets were used in the analysis to account for the under- or over-sampling of some subgroups relative to others in the dataset.

#### Study variables

The outcome variable in the study is “3+ parity”, which is defined in terms of the number of additional living children a woman has after her first two living children.

To assess the effect of the individual and socio-contextual attributes on 3+ parity, two groups of attributes are included in the analysis, namely the individual level attributes and the area level attributes.

#### Individual level attributes

Literature on individual determinants of fertility highlight five main determinants of high fertility. These are: age at first marriage, education, experience of child mortality, use and access to family planning services and income [11–17]. The individual level determinants in the current study include seven variables. These are: 1) age measured as a categorical variable (30–34 years = 0, 35–39 years =1, 40–44 years = 2, 45–49 years = 3); 2) education measured as a dichotomous variable: “less than secondary education” (1 = respondent has less than secondary education, 0 = otherwise); 3) husband’s educational level measured as a dichotomous variable: “husband’s less than secondary education” (1 = husband has less than secondary education, 0 = otherwise); 4) “proxy to household standard of living” measured as dichotomous (household does not have modern toilet facility = 1, 0 = otherwise); 5) “age at first marriage” measured as a dichotomous variable (1 = marriage at age less than 18 and 0 = otherwise); 6) “child mortality” measured as a dichotomous variable (1 = respondent had at least one child that died, 0 = otherwise); and 7) “unmet need” measured as a dichotomous variable (1 = respondent reports unmet need for spacing or limiting, 0 = otherwise). Unmet need is defined as respondent indicating desire to postpone or limit childbirth but not currently using any contraceptives.

The current study aims to assess wealth-related inequality in 3+ parity. Wealth is assessed in terms of the wealth index. The wealth index is a composite measure of a household’s cumulative living standard commonly developed in the Demographic and Health Surveys [19].

#### Area level attributes

To assess the effects of the socio-contextual factors on 3+ parity, the current study developed three scales for each area in the study. Two scales were constructed to capture the area’s socio-contextual attributes and one to assess the health system’s performance. The three scales were calculated for each area using information on all ever-married women aged 15–49 years living in this area in the EDHS dataset.

Following the Macintyre (1997) framework and its application in Judd et al. (2006) [20, 21], the first socio-contextual scale was a gendered context collective scale that measured the socio-cultural environment in which women live. Understanding that some cultural practices and norms within Egyptian communities, namely female genital cutting (FGC), early marriage, gender-based violence, and educational denial, can constrain women from achieving their potential, the scale was developed as the average of eight proportions at the level of the areas. The first five proportions assessed the appropriate gendered practices and included: 1) the proportion of women who had never experienced FGC; 2) the proportion of women who were not married before the age of 18; 3) the proportion of women who never experience any type of violence from their husband; 4) the proportion of women who never experience any violence from other people; and 5) the proportion of women who attained secondary and higher education. The remaining three proportions assessed the appropriate gendered attitudes and included: 1) the proportion of women who do not believe that FGC is required by religion; 2) the proportion of women who cannot justify spousal violence for any reason; and 3) the proportion of women who believe that the appropriate age for marriage for girls is 18 years and older.

The second scale was the contextual environmental development scale. It aimed to measure the area level environmental services. These services were assessed in terms of the proportion of women who live in households with modern toilet facilities within each area.

A health system scale was formulated to assess the effect of the health system performance on women’s 3+ parity. The scale adopted the proposed components of the World Health Organization (WHO) framework for monitoring and evaluation of health systems strengthening [22] with a particular focus on family planning. In this framework, WHO proposed the use of the contraceptive prevalence rate. The current scale added two more proportions to adequately assess the health sector’s performance on family planning, namely the proportion of met needs and the proportion of women receiving postnatal care. The latter figure was added to capture the best timing for providing counselling to women on family planning. The scale was developed as the average of three proportions, including: 1) the proportion of women currently using contraceptives; 2) the proportion of women with met needs for contraceptives; and 3) the proportion of women who received postnatal care after a delivery in the past 2 years.

To capture the effect of the low area level factors on 3+ parity, all scales were further classified into four quartiles and the analysis used the lowest quartile of each scale as a dichotomous variable (lowest quartile = 1, otherwise = 0).

### Statistical analysis

Inequality in the current study is measured using the concentration index. Since the decomposition of the concentration index requires the specification of a linear additive regression model of the health measure (h_i_) on its determinants and the current study is exploring the contribution of both the individual and the area level factors to 3+ parity inequality, the analysis implements the multilevel linear models.

#### The concentration index

The concentration index is a fairly standard measurement tool for assessing inequality in health and health care [23–25]. It allows the measurement of health inequality while taking in consideration the distribution of the health variables across all categories of the health stratifier.

For micro-level data, a convenient formula for the concentration index is defined as follows:

$$ \mathrm{CI}=\frac{2}{\upmu}\operatorname{cov}\ \left(\mathrm{h},\mathrm{r}\right), $$

where “cov” is the covariance between the health variable (h) and the fractional rank of the stratifying variable distribution. The concentration index varies between − 1 and 1 with zero indicating equality and negative values indicating disproportionate concentration of the health issue among the lowest social stratum compared to the other social strata in the population. In other words, a negative value of the concentration index for “bad” health (ill health) indicates that lower social groupings are more afflicted with this “bad” health.

#### Decomposing the concentration index

Decomposition of the health concentration index to the contributions of its main determinants requires the specification of a linear additive regression model of the health measure (h_i_) on its determinants such as:

$$ {\mathrm{h}}_{\mathrm{i}}=\upalpha +\sum \limits_{\mathrm{k}.\mathrm{i}}{\upbeta}_{\mathrm{k}}{\mathrm{x}}_{\mathrm{k}\mathrm{i}}+{\upvarepsilon}_{\mathrm{i}}, $$

where the “x_k_” variables are health determinants and ε is a disturbance term. Given the linear regression model, the concentration index for h, CI, can be written as:

$$ \mathrm{CI}=\sum \limits_{\mathrm{k}}\frac{\left({\upbeta}_{\mathrm{k}}{\overline{\mathrm{x}}}_k\right){\mathrm{C}}_{\mathrm{k}}}{\upmu}+\frac{\mathrm{G}{\mathrm{C}}_{\mathrm{e}}}{\upmu}, $$

where μ is the mean of h_i_, ${\overline{\mathrm{x}}}_k$ is the mean of *x*_*k*_, *C*_*k*_ is the concentration index for *x*_*k*_ and GC_e_ is the generalized concentration index for e_i_. In other words, the concentration index of a health measure can be divided into two main components, namely the estimated health inequalities due to inequalities in the investigated determinants, and a residual component, which captures the inequality in health that is not explained by the inequalities in these determinants.

The predicted health inequality can be simply defined as the weighted sum of the inequality in each of its determinants:

$$ \hat{\mathrm{C}\mathrm{I}}=\sum \limits_{\mathrm{k}}{\hat{\upeta}}_k{\mathrm{C}}_{\mathrm{k}} $$

The weights in this case are defined as the elasticity of the health determinant (x_k_):

$$ {\hat{\upeta}}_k=\frac{\left({\hat{\beta}}_k{\overline{\mathrm{x}}}_k\right)}{\upmu}, $$

Accordingly, for each determinant, its contribution to inequality is conceptually divided into two components: 1) the impact of the health determinant on the health measures assessed by the health elasticity (η_k_), in particular the value of the $\hat{\upbeta_{\mathrm{k}}}$; and 2) the health determinant’s unequal distribution across the socioeconomic stratifier assessed in terms of its concentration index (C_k_).

To identify the contribution of each health determinant (in percentage points) to the predicted health issue inequality, the partial contribution of each health determinant is divided by the $\hat{\mathrm{CI}}$ and multiplied by 100:

$$ \% contribution\ of\ determinant\ (K)=\frac{{\hat{\upeta}}_k{\mathrm{C}}_{\mathrm{k}}}{\hat{\mathrm{C}\mathrm{I}}}X100=\frac{{\hat{\upeta}}_k{\mathrm{C}}_{\mathrm{k}}}{\sum \limits_{\mathrm{k}}{\hat{\upeta}}_k{\mathrm{C}}_{\mathrm{k}}\ }X\ 100 $$

#### The random slope linear multilevel analysis

The multilevel modeling is commonly used for hierarchical data structure in which units in the low level are embedded within the second level units (women within areas) [26]. The multilevel modeling allows the simultaneous analysis of data for both the individual and area level variables. The random slope model allows for different relationships between an individual level variable and the dependent variable for the different areas. The general form of the multilevel random slope linear regression model is as follows:

$$ {Y}_{ij}={\gamma}_{00}+\sum \limits_P{\gamma}_{p0}{X}_{pij}+\sum \limits_q{\gamma}_{q0}{Z}_{qj}+\sum \limits_q\sum \limits_P{\gamma}_{pq}{Z}_{qj}{X}_{pij}{e}_{ij}+\sum \limits_P{U}_{pj}{X}_{pij}+{U}_{0j}+{e}_{ij} $$

Where

*i* is subscript for individual women

*j* is subscript for the community

*Y*_*ij*_ is the 3+ parity measure

*X*_*pij*_ are the woman level attributes. In the current paper, these are the woman’s age as control, marriage duration, having less than secondary education, husband has less than secondary education, proxy of household poor living condition, marrying at an age below 18, experience of child death and having unmet need.

*Z*_*qj*_ are the area level attributes. These are living in areas classified in the lowest quartile of the environmental development scale, gendered context scale and health system scale.

*γ*_00_, *γ*_*p*0_, *γ*_*pq*_ are the regression coefficients

*U*_*pj*_, *U*_0*j*_ are the residuals at the area level

*e*_*ij*_ are the residuals at the individual level

It is worth noting that marriage duration of 30–39 years is capturing in part the long duration of exposure to women who were married at a young age. With 49 years as the upper boundary for women’s age in the sample, 30–39 years of marriage meant that women in this marriage duration category were married before the age of 19. Hence, interpretation of this category was added to the effect of early marriage on 3+ parity, representing the elongation of exposure to pregnancy risk.

Due to the possible variation in the experience of early marriage age (< 18 years) across the different areas, a random slope model was assumed for early marriage across the different areas. The random effects and improvement in model fit were compared across all models by evaluating level-2 variance, change in level-2 variance, level-1 variance, intra-class correlations (ICC), and deviance (− 2 res LL) across models.

## Results

Table 1 presents a summary descriptive of the study sample. Almost 57% of those women were less than 40 years. Almost 44% of those women had never attended school or had dropped out before secondary education, and 37.7% of their husbands also had less than secondary education. More than two fifths of women live in households with no access to a modern toilet, and they were equally divided among the five quintiles of wealth. About 29% of them were married before age 18, 13.4% experienced child death during their reproductive lives, and 9.7% had unmet need. At the areas level, about one fifth of them live in areas not well served environmentally and by the health system, defined in terms of falling in the lowest quartile of both scales, and 30% of them were living in areas that adopt negative attitudes towards women.

**Table 1:** Weighted percentage distribution of individual and area attributes in the analysis (EDHS 2014)

Attributes	%
**Individual level attributes**	
Age	
30-	29.9
35-	27.0
40-	22.1
45–49	21.0
Woman’s education attainment less than secondary	43.6
Husband’s educational attainment less than secondary	37.7
Proxy for standard of living (not having modern toilet facility)	41.4
Wealth index	
Poorest	19.9
Poorer	20.6
Middle	19.3
Rich	19.4
Richest	20.8
Age at first marriage < 18 years	28.7
Experienced child death	13.4
Unmet need	9.7
**Area level attributes**	
Lowest quartile of the environmental development scale	21.2
Lowest quartile of the gendered context scale	29.9
Lowest quartile of the health system scale	21.7

Table 2 presents the mean number of 3+ parity for women aged 30 and older by the different individual and area level attributes. It shows that women with at least two living children has on average 1.52 “3+ parity” with a standard deviation of 0.013 child. In comparison to the reference category for each attribute, the mean of 3+ parity was significantly higher among women who had less than secondary education, whose husbands had less than secondary education, who have a low standard of living, who married early (below 18), who experienced child death and who had unmet need. By contrast, the mean 3+ parity was significant and positively related to age.

**Table 2:** Average 3+ parity by its different determinants (EDHS 2014)

Attributes	Mean	(STD)
**Total**	1.52	(0.014)
**Individual level attributes**		
Age ***		
30-	1.07	(0.017)
35-	1.46	(0.021)
40-	1.73	(0.026)
45–49	2.02	(0.032)
Educational attainment***		
Less than secondary	1.94	(0.021)
Secondary or more	1.19	(0.012)
Husband’s educational attainment***		
Less than secondary	1.85	(0.023)
Secondary or more	1.32	(0.013)
Proxy for wealth***		
Not having modern toilet facility	1.93	(0.021)
Having modern toilet facility	1.23	(0.014)
Wealth index***		
Poorest	2.20	(0.033)
Poorer	1.75	(0.029)
Middle	1.41	(0.026)
Rich	1.30	(0.024)
Richest	0.94	(0.017)
Age at first marriage***		
< 18 years	2.22	(0.026)
18 years and older	1.24	(0.012)
Experienced child death***		
No death	1.44	(0.012)
At least one death	2.02	(0.040)
Unmet need***		
No	1.49	(0.013)
Yes	1.79	(0.044)
**Area level attributes**		
Environmental development scale***		
Lowest quartile	2.21	(0.029)
Otherwise	1.33	(0.013)
Gendered context scale***		
Lowest quartile	2.12	(0.028)
Otherwise	1.26	(0.012)
Health system scale***		
Lowest quartile	2.35	(0.029)
Otherwise	1.29	(0.012)

*** significant at α < 0.001

At the area level, women living in disadvantaged areas that fell in the lowest quartile of each of the two socio-cultural area level attributes had significantly higher average 3+ parity compared to women living in other communities. Similarly, women living in areas falling in the lowest quartile on the health system scale showed significantly larger 3+ parity compared to women living in other areas.

The negative relationship between wealth and 3+ parity was also confirmed by the high level of the concentration index for 3+ parity (CI = − 0.165) (95% Confidence Interval = − 0.173 ~ − 0.157), with high concentration of 3+ parity among those with a lower standard of living compared to others.

### Multilevel analysis

The results of the multilevel linear regression models for the 3+ parity are presented in Table 3. It shows four nested models starting with the null model. The null model (Model 1) showed that 3+ parity was indeed clustered at the area level, with a statistically significant level-2 variance of 0.304 (SE: 0.062). The intra-class correlation coefficient (ICC), or percent of variance attributable to level 2, was 16.7% of the total variance in the model. This model showed that on average women aged 30 years and older in Egypt across all areas have 1.62 3+ parity. Model 2 included individual level attributes. The reference group in Model 2 (Table 3) were women aged 30–34 years old, both the woman and her husband having secondary education or more, belonging to a high standard of living, married at age 18 or above, having no unmet need and having never experienced child death. For this group, which represents the most advantaged socioeconomic group, the average 3+ parity is 0.76 child. Overall, Model 2 accounted for 48.4% of the level 2 variance and showed a decline in ICC to 11.1%. This result indicates significant differences in women’s individual composition across the different areas.

**Table 3:** Beta coefficients for random intercept and random slope models assessing the association between 3+ parity and individual and area level attributes (EDHS 2014)

Attributes	Model 1	Model 2	Model 3	Model 4
Constant	1.62^***^	0.763^***^	0.548^***^	0.553^***^
**Individual level attributes**				
Age				
35–39		0.435^***^	0.434^***^	0.434^***^
40–44		0.668^***^	0.668^***^	0.665^***^
45–49		0.857^***^	0.858^***^	0.855^***^
Having less than secondary education		0.221^***^	0.220^***^	0.221^***^
Husband with less than secondary education		0.057^Ϯ^	0.054^*^	0.061^*^
Low standard of living		0.164^***^	0.146^***^	0.142^***^
Marriage age < 18		0.688^***^	0.687^***^	0.710^***^
Experienced child death		0.088^*^	0.085^*^	0.080^*^
Having unmet need		0.186^***^	0.182^***^	0.180^***^
**Area level attributes**				
First quartile of gendered context scale			0.054^ns^	0.097^ns^
First quartile of environmental context scale			0.268^*^	0.184^Ϯ^
First quartile of health system scale			0.537^***^	0.495^***^
**Random effect**				
Level-2 variance	0.304 (0.062)	0.157 (0.044)	0.042 (0.014)	0.032 (0.010)
∆ (%) level-2 variance	–	48.4%	86.2%	89.5%
Variance random slope (marrying at age less than 18 years)				0.067 (0.021)
Level-1 variance	1.521 (0.107)	1.247 (0.078)	1.248 (0.078)	1.237 (0.078)
Intra-class correlation	16.7%	11.2%	3.3%	2.5%
Log likelihood	−18,983.449	−17,819.711	−17,792.046	−17,767.053

*** significant at α < 0.001; * significant at α < 0.05; Ϯ significant at α < 0.10

Overall, Model 2 revealed, as expected, that age was linearly and positively associated with 3+ parity. Older women were more likely to have higher 3+ parity than young women and those who had been married for a short duration. Women with less than secondary education (β = 0.22), living in a household with a low standard of living (β = 0.16), marrying at an early age (< 18 years) (β = 0.69), having an unmet need (β = 0.19) and experienced a child death (β = 0.09) were all positively and significantly associated with 3+ parity. By contrast, marrying a husband with less than secondary education was marginally associated with 3+ parity.

Model 3 introduced the area level attributes. Model 3 captured 86.2% of the level 2 variance in 3+ parity. It showed a decrease in ICC from 11.1 to 3.3%, indicating the ability of the added area indicators to explain a significant part of the area variability in 3+ parity. Model 3 also showed that the addition of the area level attributes had no effect on the coefficients of the individual level attributes. For the area level attributes, living in areas in the lowest quartile on the health system scale showed the highest significant positive effect on 3+ parity among three areas’ level attributes. Living in these areas increased 3+ parity by 0.54 children. Living in the areas falling in the lowest quartile of the environmental context also had significant positive effect on 3+ parity, while living in areas falling in the lowest quartile of the gendered context scale had no significant effect on 3+ parity with an increase of (0.05) in 3+ parity.

Model 4 examined the effect of assuming differences in the experience of early marriage across the different areas. The log likelihood ratio test statistics showed a decrease of 25 points, indicating very strong evidence for the hypothesized variation of early marriage across the different areas. This model showed a decrease in the level-2 variance by 89.5%, while ICC reached 2.5%. Model 4 showed only slight changes in the coefficients of the individual level attributes. Allowing early marriage to differ across the areas decreased the coefficients for the area level attributes. The largest change was in the coefficient of living in the areas falling in the lowest quartile environmental context scale, which declined from 0.268 to 0.184 between Model 3 and Model 4 and became marginally related to 3+ parity. The coefficient for living in areas falling in the lowest quartile in the health system scale also declined from 0.537 in Model 3 to 0.495 in Model 4. The least change was in the coefficient of living in the areas that fell in the lowest quartile of the gendered context scale. The coefficient increased from 0.054 in Model 3 to 0.097 in Model 4, but continue to have no significant relation to 3 + parity.

Overall, the final model succeeded in accounting for almost all the level-2 variance and confirmed the differential effect of early marriage across the communities on 3+ parity.

### Decomposition of inequality in 3+ parity

Using the results of the final model (Model 4), the decomposition analysis was carried out on both the fixed effects part and the random effects part of the model. While the fixed effects part of the model focuses on the average relationship between 3+ parity and both the individual and area level attributes, the random effects part moves forward to examine the varying effects of early marriage among the different areas on 3+ parity.

Overall, the concentration index for the predicted 3+ parity in the final model showed a high concentration among poor women ($\hat{\mathrm{CI}}$ = − 0.141), which accounted for 85.4% of overall sample concentration index (CI = − 0.165). The decomposition analysis showed that the concentration index of the predicted 3+ parity was split into ($\hat{\mathrm{CI}}$ = − 0.131) for the fixed effect part of the multilevel model and ($\hat{\mathrm{CI}}$ = − 0.010) for the random effects part of the multilevel model, representing 92.9 and 7.1% of $\hat{\mathrm{CI}}$, respectively. Furthermore, the decomposition of the fixed effects part of the model shows that $\hat{\mathrm{CI}}$ for the fixed effect part can be divided into ($\hat{\mathrm{CI}}=-0.0785$) for the individual level attributes and $\hat{\Big(\mathrm{CI}}=0.0521\Big)$ for area level attributes representing 55.7 and 37% of overall inequality in 3+ parity, respectively. In other words, inequality in individual level attributes was responsible for 55.7% of the inequality in 3+ parity, leaving 44.3% for the inequality in area level attributes.

Focusing on the contribution of the individual level attributes, Table 4 shows that the highest share of the wealth-based inequality in 3+ parity was mainly attributed to early marriage. The early marriage attribute showed a contribution of 25.9% of $\hat{\mathrm{CI}}$ for 3+ parity. This large share was the product of a large effect of early marriage on 3+ parity (β = 0.71) and the high concentration of the poor in this attribute (CI for early marriage = − 0.287).

**Table 4:** Contribution of individual and community level determinants based on the decomposition of concentration index analysis for 3+ parity (EDHS 2014)

Attributes	Coef.	Average	Elasticity	CI	Contribution to CI	% Contribution
	Full model					
Predicted high parity	–	1.585			− 0.126	
**Individual level attributes**						
Age (ref:30–34)						
35–39	0.434	0.270	0.077	0.009	0.001	−0.5
40–44	0.665	0.221	0.097	− 0.005	0.000	0.3
45–49	0.855	0.210	0.118	−0.009	− 0.001	0.7
Having less than secondary education	0.221	0.436	0.063	−0.286	−0.018	12.8
Husband with less than secondary education	0.061	0.377	0.015	−0.235	−0.004	2.5
Low standard of living	0.142	0.414	0.039	−0.438	−0.017	12.0
Marriage age < 18	0.710	0.287	0.134	−0.272	−0.036	25.9
Experienced child death	0.080	0.134	0.007	−0.233	−0.002	1.2
Having unmet need	0.180	0.096	0.011	−0.094	− 0.001	0.8
**Area level attributes**						
First quartile of gendered context scale	0.097	0.299	0.019	−0.426	− 0.008	5.8
First quartile of environmental context scale	0.184	0.212	0.026	−0.491	−0.013	8.9
First quartile of health system	0.495	0.217	0.071	−0.445	−0.031	22.3

Women having less than a secondary education captured another 12.8% of $\hat{\mathrm{CI}}$ for 3+ parity. This attribute showed moderate effect on 3+ parity (β = 0.22) but exhibited high inequality with a high concentration of less than secondary education among the poor (CI = -0.29). The proxy for the women’s household standard of living also accounted for 12.0% of $\hat{\mathrm{CI}}$ for 3+ parity. A low standard of living was not responsible for large increases in 3+ parity (β = 0.14), but there was high concentration of poor women in the low standard of living category (CI = -0.44). Other attributes accounted for less than 3% of the 3+ parity inequality. Unmet need exhibited low contribution to $\hat{\mathrm{CI}}$ for 3+ parity, accounting for less than 1% of it. Although it was significantly related to high parity (β = 0.19), it showed low wealth-based inequality (CI = -0.094). For experience of child death, the contribution was also minimal due to its small effect on 3+ parity, despite its high concentration among the poor (CI = -0.233).

For the share in the fixed effect part, living in areas falling in the lowest quartile on the health system scale accounted for 22.3% of $\hat{\mathrm{CI}}$ for 3+ parity. This large contribution is the product of the large effect of living in these areas on 3+ parity (β = 0.495) and the high concentration of the poor in them (IC = -0.44).

Living in areas classified in the lowest quartile of the gendered context scale also contributed 5.8% of $\hat{\mathrm{CI}}$ for 3+ parity. Although living in these areas had a small positive effect on 3+ parity (β = 0.097), high concentration of the poor in these areas (IC = -0.43) was largely responsible for its contribution to $\hat{\mathrm{CI}}$ for 3+ parity. Living in environmentally underserved areas contributed only 8.9% of $\hat{\mathrm{CI}}$ for 3+ parity. This contribution was the product of its relatively moderate effect on 3+ parity (β = 0.18), but high concentration of the poor in them (CI = -0.49).

For the random effects part of the model, focusing on the contributions of the specific areas, the (− 0.010) was decomposed among the 47 areas and the early marriage attribute within these 47 areas. Table 5 showed the details of the decomposition analysis for the top five areas that contribute to $\hat{\mathrm{CI}}$ for 3+ parity. The highest contribution was found in living in Cairo, urban Alexandria, rural Assiut and rural Sharkia and Gharbia. These areas accounted for 13.9% of $\hat{\mathrm{CI}}$ for 3+ parity. These areas exhibited two patterns of contributions. Cairo, urban Alexandria and rural Gharbia exhibited relatively large negatively effects on 3+ parity (β = − 0.19, − 0.33, − 0.17, respectively) and these areas housed a significantly low concentration of poor women (CI = 0.69, 0.64 and 0.59, respectively). This resulted in contributions of the value 5.5, 4.5%, and .9%, respectively. By contrast, living in rural Assiut and Sharkia was positively related to 3+ parity (β = 0.284) and exhibited a high concentration of poor women (CI = -0.537) which resulted in a contribution of 3.1%.

**Table 5:** Contribution of top 5 areas to the decomposition of concentration index for random effects of the multilevel model of 3+ parity (EDHS 2014)

Area^a^	Coeff.	CI	Average	Contribution to CI	% Contribution
Cairo	− 0.192	0.686	0.090	−0.008	5.527
Alexandria_u	−0.337	0.640	0.044	−0.006	4.466
Assiut_r	0.245	−0.537	0.032	−0.003	1.976
Sharkia_r	0.100	−0.324	0.071	−0.002	1.075
Gharbia_u	−0.172	0.595	0.019	−0.001	0.901

^a^ _u and _r refer to urban and rural areas within the indicated governorate, respectively

For the effect of early marriage within the different areas, Table 6 shows the details of the decomposition for the top five contributing areas. It showed that living in rural Minya, rural Sohag, rural Beni Suef and Cairo, and marrying at an age below 18 years, contributed 3.5% of $\hat{\mathrm{CI}}$ for 3+ parity. Living in the first three areas and rural Qena; and marrying at less than 18 years were positively related to 3+ parity (β = 0.23, 0.17, 0.13, and 0.13 respectively). In addition, there are high concentration of poor women among those who were in this situation (− 0.64, − 0.73, − 0.58 and − 0.53, respectively). By contrast, living in Cairo was negatively related to 3+ parity (β = − 0.136) and there was less concentration of poor women living in Cairo and marrying at less than 18 years (CI = 0.554).

**Table 6:** Contribution of top 5 areas for early marriage within area based on the decomposition of concentration index for random effects of the multilevel model of 3+ parity (EDHS 2014)

Area^a^	Coeff.	CI	Average	Contribution to CI	% Contribution
Minya_u	0.234	−0.640	0.022	−0.002	1.546
Beni Suef_r	0.166	−0.582	0.012	−0.001	0.532
Sohag_r	0.130	−0.733	0.012	−0.001	0.514
Cairo	−0.136	0.554	0.014	−0.001	0.496
Qena_r	0.130	−0.533	0.012	−0.001	0.378

^a^ _u and _r refer to urban and rural areas within the indicated governorate, respectively

## Discussion

Population growth and its negative impact on the expected returns of many years of development and on economic reform efforts in Egypt have been the subject of significant political commitment. However, in a country with limited resources like Egypt, efficient use of these resources and identification of the most influential points of intervention are two imperative criteria to curb fertility. Embracing a two-child ideal and decreasing the intensity of transitioning to the third child have been documented as playing a major role in countries in the same transitional stage as Egypt [12]. While previous literature focused on identifying the attributes of the “pioneer” social subgroups, only limited research has explored the attributes of the laggers who exceeded the replacement fertility level of two children per family.

The analysis in the current study investigates 3+ parity among women in Egypt, while taking into consideration the effect of both individual and area level factors. At the individual level, the analysis confirms previous research showing the positive effects of living in a low standard of living household, having low levels of education and early age of marriage on 3+ parity [11–17]. It also confirmed previous research that indicated the weakening of the education effect against the age at marriage [17]. While having less than secondary education increases 3+ parity by 0.22, early age at marriage increases parity by 0.71. The study also confirmed the positive effect of unmet need in increasing levels of 3+ parity [27].

In reviewing the main determinants of high fertility, Casterline (2010) pointed out the importance of the area level attributes in terms of obstacles to contraception or family planning services in shaping high fertility. He further stated that for the socio-contextual factors, “Obstacles to contraception: Non-access obstacles (cultural, social, psychic) appear to be robust in some settings but are not well quantified,” and for family planning services, “the evidence on access obstacles is less ambiguous: in diverse settings expanded provision of family planning services has had an impact on fertility, typically 10%–25% net reduction in fertility” [22]. The current analysis was able to quantify the significant contribution of the area level attributes to explaining 3+ parity. Area level factors explained more than 16% of the 3+ parity variability. The results also confirmed the significant role of the health system. Living in area with a low level of health system performance contributed an addition of more than 0.5 a child, which represents 14% of the current TFR in Egypt.

From a policy perspective, these results call for an intervention targeted at the individual level attributes and the community level attributes for the health system, which is the strategy that has been adopted in Egypt for a long time. Addressing 3+ parity policies focuses on tackling early age at marriage, raising women’s educational attainment to secondary education, adopting poverty alleviation interventions and satisfying unmet need, as well as strengthening the health system for provision of efficient and adequate family planning services.

A novel contribution of the current study is the introduction of the inequality decomposition analysis of the multilevel modeling of 3+ parity. This contribution recognized the high concentration of 3+ parity among poor women. It allowed the identification of the main contributors to this inequality. Guided by the results of this analysis, policy makers can define the main points of intervention that tackle this inequality and thus address the high level of fertility in Egypt.

The results of the decomposition analysis revealed that 55.7% of the 3+ parity inequality were attributed to individual level attributes and 44.3% were attributed to area level attributes. In other words, while the explanation effect of the area level attributes accounted for less than 9% of the variability in 3+ parity, controlling for the individual level attributes, being poor and living in specific areas contributed 46.8% of the 3+ parity inequality. Accordingly, tackling this inequality calls for actions and interventions in relation to both the individual and area level attributes.

From a policy perspective, the decomposition analysis at the individual level confirmed the regression results and previous literature with regard to early age at marriage. Early marriage accounted for 26% of the inequality in 3+ parity. Since marriage is the only track through which women can have children, early marriage expands women’s exposure to pregnancy risk for a long period. This prolonging of exposure, combined with the high concentration of early marriage among the poor, calls for two types of policies and interventions. The first type focuses on tackling early marriage with particular emphasis on its high concentration among poor women, while the second type of intervention focuses on women who marry early and attempts to attenuate the positive effect of early marriage on 3+ parity.

About 13% of the 3+ parity inequality were due to low levels of education and attribute to 3+ parity inequality were mainly due to its high positive effect on 3+ parity. Policies in this case should focus on interventions that attenuate the effect of low levels of education on 3+ parity and which simultaneously work towards retaining poor women in the education system in order to complete secondary education. Poverty is key in eliminating inequality. Nevertheless, poor households should also receive intensive support, as they account for 12% of 3+ parity inequality.

For policies at the area level, the decomposition analysis stressed the effect of the health systems performance. It suggests that tackling 3+ parity inequality requires improving the health system in the areas with low family planning services beyond the threshold of the lowest quartile level of the health system scale, as well as intervening to improve the living conditions of poor women residing in these areas through tailored developmental efforts targeting those women. Similarly, highly gendered areas call for interventions to address these gendered attitudes and their positive effect on 3+ parity, and at the same time work to improve the living condition of poor women in these areas.

The decomposition analysis also allowed the identification of the specific contribution of some areas to 3+ parity inequality. The privileged conditions of living in Cairo (the capital), urban Alexandria (the second largest metropolitan area in Egypt), and urban Gharbia contributed 11.1% to 3+ parity inequality through their negative effect on 3+ parity, as well as the low concentration of poor women in these areas. By contrast, the deprived areas, in particular rural Assiut, contributed 2.8% to 3+ parity inequality through their positive relationship with 3+ parity and the high concentration of poor people living in those areas. Interventions in this case should involve studying and identifying the main factors that promoted low parity in the first three communities and the low concentration of poverty and adopting them in the other communities. For rural Assiut, interventions should be directed to identify the other factors that led to a positive effect on 3+ parity and adopting more targeted development efforts to reduce the high concentration of poor people in this community.

Focusing on the combined effect of early marriage in specific areas revealed that the combination of living in the rural areas in Minya, Sohag, Beni Suef, and Qena and marrying at an early age contributed 3.5% to 3+ parity inequality. Policy interventions should focus on identifying the other factors that led to these areas’ positive effect on 3+ parity and implementing more targeted development efforts to reduce the high concentration of poor and early married women in these regions.

## Conclusion

The current study introduces a comprehensive approach to support policy interventions to address high parity in Egypt. It started by highlighting the importance of taking the extra step towards the investigation of the area level, in a manner that takes into consideration the clustering of the individuals within areas. It also introduced a decomposition analysis of both the individual and area level attributes. Reliance on health modeling has proven inefficient in the identification of the main determinants of 3+ parity inequality. The use of multilevel decomposition allowed the quantification of the contributions of both the individual and area level attributes. The decomposition also allowed the identification of detailed policy implications and solutions to tackle inequality. Although poverty alleviation policies are important, the current study calls for more tailored policies that target different social groups and different areas. At the area level, in addition to identifying the contribution of cross cutting area attributes, the analysis was able to identify specific areas that warrant extra attention and concerns. This targeted approach can be used to support the efficient use of limited resources within any society.

## Data Availability

The dataset analyzed during the current study is available in The DHS program: The Demographic and Health Survey program repository. https://www.dhsprogram.com/data/dataset/Egypt_Standard-DHS_2014.cfm?flag=0

## Abbreviations

CI: Concentration index
DHS: Demographic and health survey
EDHS: Egypt demographic and health survey
CSDH: Commission on Social Determinants of Health
